# Curcumin and Plumbagin Synergistically Target the PI3K/Akt/mTOR Pathway: A Prospective Role in Cancer Treatment

**DOI:** 10.3390/ijms24076651

**Published:** 2023-04-02

**Authors:** Iftikhar Ahmad, Mehboob Hoque, Syed Sahajada Mahafujul Alam, Torki A. Zughaibi, Shams Tabrez

**Affiliations:** 1 Department of Biochemistry, Faculty of Science, King Abdulaziz University, Jeddah 21589, Saudi Arabia; 2 King Fahd Medical Research Center, King Abdulaziz University, Jeddah 21589, Saudi Arabia; 3 Applied Bio-Chemistry Laboratory, Department of Biological Sciences, Aliah University, Kolkata 700160, India; 4 Department of Medical Laboratory Sciences, Faculty of Applied Medical Sciences, King Abdulaziz University, Jeddah 21589, Saudi Arabia

**Keywords:** cancer therapy, curcumin, MD simulation, molecular docking, plumbagin, synergism

## Abstract

Cancer development is associated with the deregulation of various cell signaling pathways brought on by certain genetic and epigenetic alterations. Therefore, novel therapeutic strategies have been developed to target those pathways. The phosphoinositide 3-kinase (PI3K)/protein kinase B (Akt)/mammalian target of rapamycin (mTOR) (PI3K/Akt/mTOR) pathway is one major deregulated pathway in various types of cancer. Several anticancer drug candidates are currently being investigated in preclinical and/or clinical studies to target this pathway. Natural bioactive compounds provide an excellent source for anticancer drug development. Curcumin and plumbagin are two potential anticancer compounds that have been shown to target the PI3K/Akt/mTOR pathway individually. However, their combinatorial effect on cancer cells is still unknown. This study aims to investigate the synergistic effect of these two compounds on the PI3K/Akt/mTOR pathway by employing a sequential molecular docking and molecular dynamics (MD) analysis. An increase in binding affinity and a decrease in inhibition constant have been observed when curcumin and plumbagin were subjected to sequential docking against the key proteins PI3K, Akt, and mTOR. The MD simulations and molecular mechanics combined with generalized Born surface area (MM-GBSA) analyses validated the target proteins’ more stable conformation when interacting with the curcumin and plumbagin combination. This indicates the synergistic role of curcumin and plumbagin against cancer cells and the possible dose advantage when used in combination. The findings of this study pave the way for further investigation of their combinatorial effect on cancer cells in vitro and in vivo models.

## 1. Introduction

Cancer ranks as the second leading cause of premature death worldwide, just after cardiovascular disease (CVD). Current trends suggest that in most countries, cancer may eventually overtake CVD as the primary cause of death [1]. Cancer comprises a group of diseases featuring uncontrolled/abnormal growth of cells, spreading all over the body via metastasis. There are multifarious factors that lead to the development and progression of cancer. Dysregulation of numerous cell signaling pathways brought on by certain genetic and epigenetic alterations is one of the most important factors that contribute to the development of cancer [2]. The phosphoinositide 3-kinase (PI3K)/protein kinase B (Akt)/mammalian target of rapamycin (mTOR) (PI3K/Akt/mTOR) is one such pathway, whose deregulation has been found to be associated with various types of human cancers [3,4,5]. The over-activated PI3K/Akt/mTOR pathway leads to the manifestation of a variety of cancer hallmarks, including growth signal autonomy, evasion of apoptosis, induction of angiogenesis, tissue invasion and metastasis, and insensitivity to antigrowth signals [6,7]. Therefore, this pathway is considered a significant target for the development of novel anticancer therapies.

Conventional cancer therapies frequently fail because of their unreliable results, negative side effects, development of drug resistance, toxic effects on healthy cells, and difficulty in accessing the target tumor tissues. Therefore, efforts are being made to minimize the adverse effects and improve therapeutic efficacy. Recent developments point to a paradigm shift towards plant-derived natural compounds for the development of safe and effective cancer treatment [5,8,9]. 

**Figure 1:**
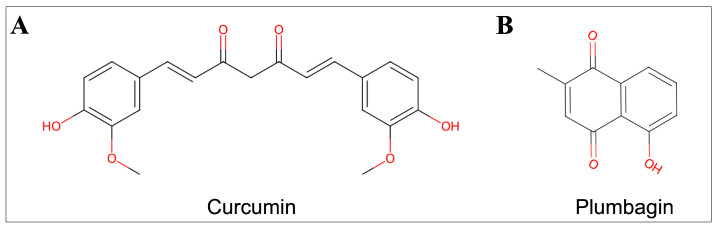
Chemical structures of (**A**) curcumin and (**B**) plumbagin.

Curcumin is the most beneficial bioactive compound derived from *Curcuma longa,* which possesses various medicinal benefits [10]. Several preclinical and clinical studies reported the potent anticancer efficacy of curcumin via targeting multiple signaling pathways, including the PI3K/Akt/mTOR pathway involved in cancer development and progression [11,12,13]. Another key natural bioactive compound, plumbagin, which is found in plants of the Plumbaginaceae family, has been greatly explored for its anticancer activity [14]. This compound has also been noted to exhibit anticancer activity by effectively targeting various cell signaling pathways, including PI3K/Akt/mTOR [15,16]. The molecular structures of curcumin and plumbagin are shown in Figure 1.

Thus, it has been demonstrated that curcumin and plumbagin both exhibit anticancer activity by inhibiting the PI3K/Akt/mTOR pathway separately. Their combined effect on this pathway has not yet been reported. Accumulating reports suggest that the combinatorial action of drugs exhibits superior efficacy for the treatment of cancer [17,18]. The present study has been designed to investigate how curcumin and plumbagin work together to target the PI3K/Akt/mTOR pathway, which may have implications in cancer therapy. Sequential molecular docking and molecular dynamics (MD) were performed to determine if the two anticancer bioactive compounds have an antagonistic or synergistic impact on the important target proteins PI3K, Akt, and mTOR.

## 2. Results and Discussion

### 2.1. Sequential Molecular Docking Determines the Synergy of Curcumin and Plumbagin

To understand the combinatorial effect of the two compounds, plumbagin and curcumin were docked individually and in combination with the three key proteins of the PI3K/Akt/mTOR pathway. Plumbagin showed a binding energy of −6.44 kcal/mol for the PI3K protein, whereas curcumin had a binding energy of −9.04 kcal/mol. For Akt protein, plumbagin had a binding energy of −6.06 kcal/mol, while curcumin had a binding energy of −8.31 kcal/mol. Plumbagin binds to the mTOR protein with a binding energy of −5.09 kcal/mol, while curcumin binds with a binding energy of −5.13 kcal/mol. As determined by individual as well as sequential docking, Table 1 shows the interactive residues between the two compounds and each target protein/protein complex under consideration.

**Figure 2:**
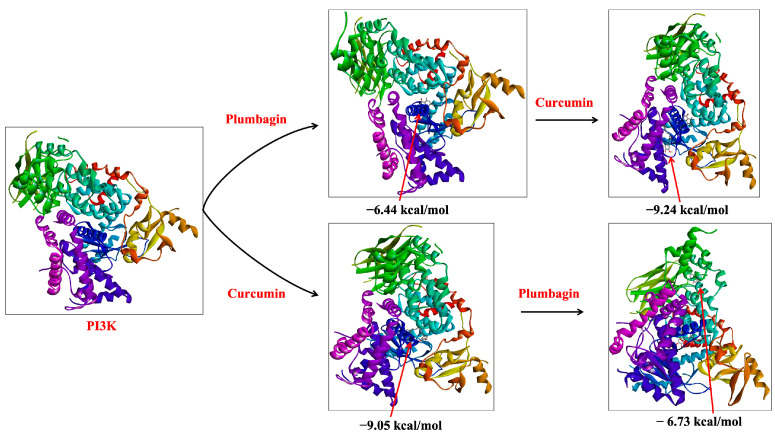
Sequential binding of curcumin and plumbagin after docking against PI3K protein. The red arrows indicate the position of bound ligand on the target protein.

**Figure 3:**
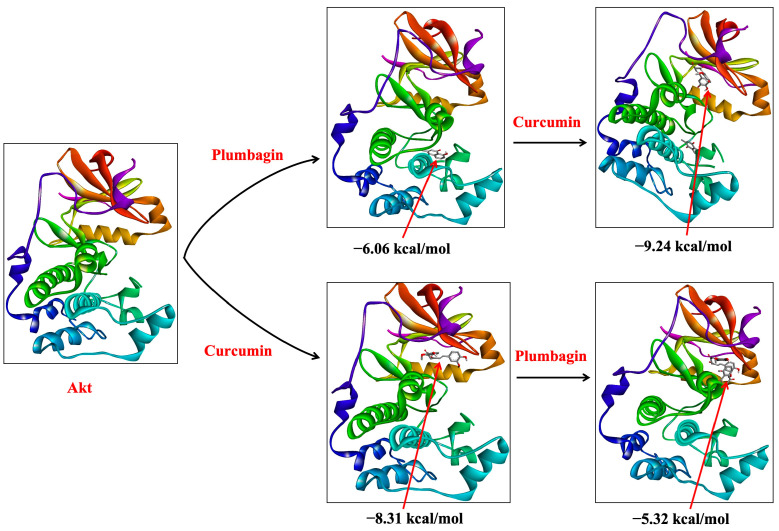
Sequential binding of curcumin and plumbagin after docking against Akt protein. The red arrows indicate the position of bound ligand on the target protein.

In our sequential docking analysis, we observed that the docking score for curcumin was increased to −9.24 kcal/mol when docked with plumbagin-associated PI3K (PI3K-P-C) from −9.05 kcal/mol with the protein PI3K alone (PI3K-C) (Table 1). Similarly, if curcumin was already associated with the protein structure of PI3K (PI3K-C), plumbagin’s docking (PI3K-C-P) score increased to −6.73 kcal/mol from the initial −6.44 kcal/mol (Figure 2). This result indicates that the binding of one ligand to PI3K protein increased the binding affinity of the other compound. The synergistic binding is also supported by the decrease in the inhibition constant (Ki) in the sequential docking (Table 1). Similar results were also observed in Akt binding studies. If plumbagin was already associated with the protein structure of Akt (Akt-P), the docking score of curcumin increased up to −9.24 kcal/mol of (Akt-P-C) (from −8.31 kcal/mol for Akt-C). However, the binding energy of plumbagin (Akt-C-P) was found to decrease marginally to −5.32 kcal/mol (from −6.06 kcal/mol) when curcumin was associated with the protein structure of Akt (Akt-C) (Table 1). These data suggest that the binding of plumbagin enhances the affinity of curcumin to Akt, indicating synergism. However, if curcumin is already bound to Akt, it might show antagonistic behavior, as they seem to compete for the binding site (Figure 3).

**Figure 4:**
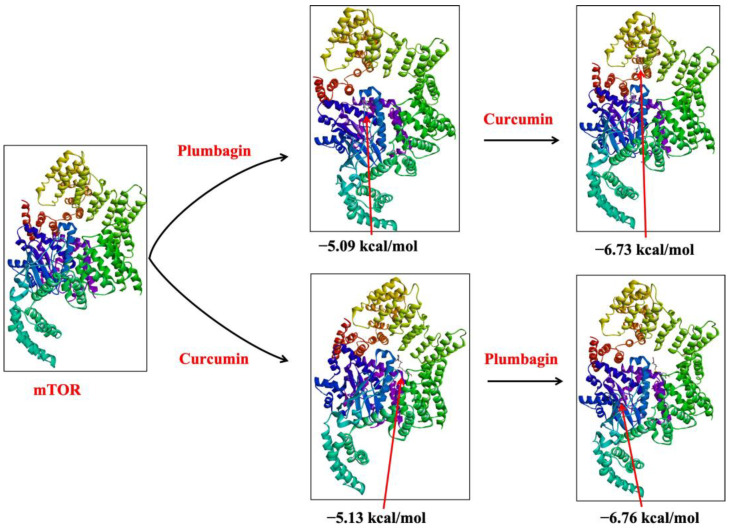
Sequential binding of curcumin and plumbagin after docking against mTOR protein. The red arrows indicate the position of bound ligand on the target protein.

Similarly, if plumbagin was already associated with the protein structure of mTOR (mTOR-P), the binding affinity of curcumin increased to −6.73 kcal/mol (Akt-P-C) from their isolated affinity of −5.13 kcal/mol (Akt-C). Likewise, if curcumin was already associated with the protein structure of mTOR (mTOR-C), plumbagin’s docking (mTOR-C-P) score increased to −6.76 kcal/mol (from −5.09 kcal/mol) (Figure 4). These findings clearly demonstrate the synergistic role of curcumin and plumbagin, which indicates that a combination of these drugs is more effective than the individual drug against the PI3K/Akt/mTOR pathway.

Recent reports suggest that the plumbagin reduces the proliferation of human bladder cancer cells both in vivo and in vitro, suggesting that it may have a role in cell cycle arrest and apoptosis induction via numerous downstream effectors of the PI3K/Akt/mTOR signaling pathway [15]. Another study suggested suppression in the PI3K/Akt/mTOR pathway, leading to induced apoptosis as a result of plumbagin treatment in rat ovarian granulosa cells [19]. In numerous tumor models, curcumin has been found to block the PI3K/Akt signal transduction pathway [11]. Curcumin, for example, has been reported to inhibit the PI3K/Akt pathway and its downstream NF-κB protein expression, thereby enhancing radiation-induced apoptosis in human Burkitt’s lymphoma cells [20]. In a separate study, Yu et al. (2008) reported that curcumin inhibited the activation of Akt/mTOR signaling in prostate cancer [21]. Moreover, in a recent study, curcumin has also been shown to inhibit head and neck cancer growth and progression by downregulating the PI3K/Akt/mTOR signaling pathway [22]. 

Overexpression of PI3K/Akt/mTOR pathways allows cancer cells to survive for longer periods. Therefore, the downregulation of this pathway plays an important regulatory mechanism in combating cancer growth and progression. The individual anticancer effect of both curcumin and plumbagin by targeting PI3K/Akt/mTOR signaling has been reported earlier. However, their combinatorial effect on pro-survival signaling has not yet been reported. The findings of this study demonstrate for the first time that these compounds possess a strong potential for a synergistic anticancer effect by targeting the PI3K/Akt/mTOR pathway.

### 2.2. MD Simulation Analysis of Curcumin and Plumbagin Targeting PI3K/Akt/mTOR

Molecular dynamics (MD) simulation is reliably used for the analysis of atomic behavior, structural stability, and conformational changes at the atomic level of a protein [23,24]. In this study, the synergistic interactions of curcumin and plumbagin with the target proteins were further investigated by performing an MD simulation. These investigations were performed to ascertain the convergence and stability of the target protein–ligand complexes of PI3K (PI3K-P, PI3K-C, and PI3K-C-P), Akt (Akt-P, Akt-C, and Akt-C-P), and mTOR (mTOR-P, mTOR-C, and mTOR-C-P). The root means square deviation (RMSD) values of individual protein and ligand docked complex were compared, and each of the simulation studies of 100 ns demonstrated steady conformation. 

**Figure 5:**
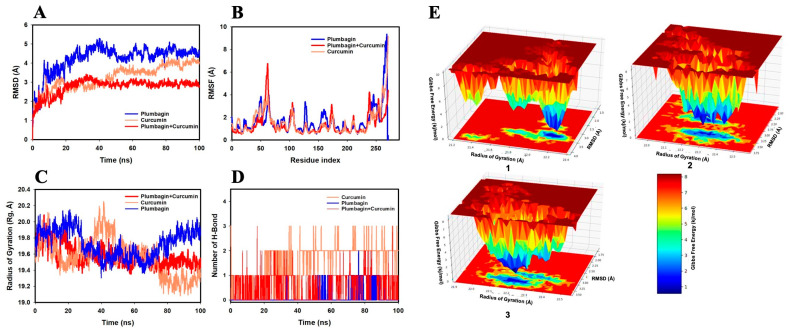
Analysis of MD simulation trajectories with a 100 ns time scale for the interaction of curcumin and plumbagin with PI3K. (**A**) Root means square deviation (RMSD) plot displaying the molecular vibration of Cα backbone of PI3K-P (blue), PI3K-C (orange), and PI3K-C-P (red). (**B**) Root mean square fluctuation (RMSF) plot showing the fluctuations in respective amino acids throughout the simulation time of 100 ns for PI3K-P (blue), PI3K-C (orange), and PI3K-C-P (red). (**C**) Radius of gyration (Rg) plot for the deduction of compactness of PI3K-P (blue), PI3K-C (orange), and PI3K-C-P (red). (**D**) Number of H-bonds formed between PI3K and plumbagin (blue), PI3K and curcumin (orange), and PI3K and curcumin–plumbagin combination (red). (**E**) The free energy landscape (FEL) displaying the progression of global minima (ΔG, kJ/mol) of PI3K in presence of (**1**) plumbagin, (**2**) curcumin, and (**3**) curcumin–plumbagin combination with respect to their RMSD (Å) and Rg (Å). The lower right panel displays the energy scale used in the measurement of FEL.

The RMSD of the Cα-backbone of PI3K bound to plumbagin (PI3K-P) showed a deviation of 4.2 Å (Figure 5A). On the other hand, the Cα-backbone of the target protein bound to curcumin (PI3K-C) showed a deviation of 3.9 Å. Interestingly, the consortia of plumbagin and curcumin bound to PI3K protein (PI3K-C-P) exhibited a very stable RMSD of 2.3 Å. A stable RMSD plot during simulation signifies good convergence and stable conformations [23,25,26,27]. Therefore, it can be suggested that PI3K forms a quite stable complex when subjected to synergistic binding with plumbagin and curcumin. Similarly, the plot for root mean square fluctuation (RMSF) displayed significant spikes of fluctuation in Cα-atoms of PI3K when bound to plumbagin or curcumin, while less-significant spikes were observed with plumbagin and curcumin-bound PI3K (PI3K-C-P) (Figure 5B). At the residues 48–69 and 240–250, higher residual fluctuations were observed. The increased flexibility of the residues conformed into the loop region may be the cause of high residual fluctuations, whereas the lower fluctuation of the other residues throughout the course of 100 ns simulation suggests rigid amino acid conformations (Figure 5B). Therefore, it can be inferred from RMSF plots that the proteins have rigid structures during simulation in ligand-bound conformations [26,27]. In this study, the radius of gyration Rg (which denotes the compactness of a protein) values of the protein C-backbone of the PI3K-P complex increased from 19.9 to 20.0 Å, but the Rg values of the PI3K-C complex decreased from 19.9 to 19.3 Å (Figure 5C). The PI3K-C-P also showed a decrease in Rg peak and finally stabilized at 19.7 Å. The lower and more consistent Rg peak indicates that the protein complex in the ligand-bound state is more compact. Moreover, the number of H-bonds formed between a protein and a ligand indicates the importance of the interaction and the complex stability. Throughout the simulation length of 100 ns, the number of H-bonds between the protein and the ligands was substantial (Figure 5D). A consistent number of H-bonds was detected between PI3K and the ligands in the PI3K-P complex, with an average of one H-bond, three H-bonds in the PI3K-C complex, and a couple of H-bonds in the PI3K-C-P complex throughout the simulation time, which may facilitate a stable complex conformation (Figure 5D).

Figure 5E depicts the free energy landscape (FEL) of attaining the global minima of Cα backbone atoms of the PI3K protein with regard to RMSD and Rg. The global minima (lowest free energy state) of the PI3K-P complex were achieved at RMSD 3.5 Å and Rg 22.2 Å (Figure 5E(1)). Because of the great stability and optimal conformation in the plumbagin-bound state (PI3K-P), the FEL anticipated the deterministic behavior of PI3K to be the lowest-energy state. In the instance of PI3K bound with curcumin (PI3K-C), the global minima were achieved at RMSD 3.25 Å and Rg 22.2 Å (Figure 5E(2)). However, the global minima for PI3K bound to the plumbagin and curcumin (PI3K-C-P) consortium were achieved at RMSD 3.0 Å and Rg 22.1 Å (Figure 5E(3)). The deep blue basin denotes the achievement of global minima at the corresponding RMSD and Rg values. The FEL is an indicator of protein folding to attain a minimum energy state, which is achieved well by the plumbagin and curcumin combination bound state.

**Figure 6:**
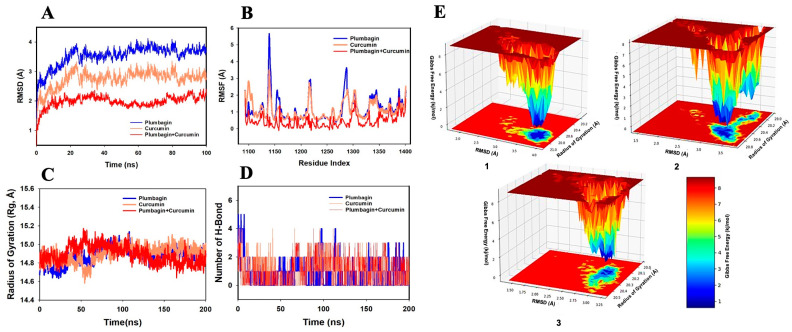
Analysis of MD simulation trajectories with a 100 ns time scale for the interaction of curcumin and plumbagin with Akt. (**A**) RMSD plot displaying the molecular vibration of Cα backbone of Akt-P (blue), Akt-C (orange), and Akt-C-P (red). (**B**) RMSF plot showing the fluctuations of respective amino acids throughout the simulation time of 100 ns for Akt-P (blue), Akt-C (orange), and Akt-C-P (red). (**C**) Rg plot for the deduction of compactness of Akt-P (blue), Akt-C (orange), and Akt-C-P (red). (**D**) Number of H-bonds formed between Akt and plumbagin (blue), Akt and curcumin (orange), and Akt and curcumin–plumbagin combination (red). (**E**) The FEL displays the progression of global minima (ΔG, kJ/mol) of Akt in presence of (**1**) plumbagin, (**2**) curcumin, and (**3**) curcumin–plumbagin combination with respect to their RMSD (Å) and Rg (Å). The lower right panel displays the energy scale used in the measurement of FEL.

While performing MD simulations with Akt ligand complexes, the RMSD values of the Cα-backbone of Akt-P and Akt-C were found to exhibit deviations of 3.7 Å and 2.7 Å, respectively (Figure 6A). In the combination of compounds, when bound to Akt protein, the complex (Akt-C-P) exhibited an RMSD value of 2.0 Å, suggesting a good convergence and a stable conformation. The RMSF plot displayed significant spikes of fluctuation in Cα-atoms of Akt-P and Akt-C complexes, while no significant spikes were observed in the Akt-C-P complex. At residues 113–1150, 1210–1225, and 1280–1300, higher residual fluctuations were observed. The higher residual fluctuations signify higher flexibility of the residues in the loop region, while the less-fluctuating residues indicate the rigid amino acid conformations during the simulation time (Figure 6B). Therefore, the RMSF plots of Akt interaction with the combination of ligands suggest a rigid protein conformation during the course of the simulation [26,27]. Furthermore, the compactness of the Akt protein was evaluated by measuring the Rg value. Here, the Cα-backbone of the Akt-P complex exhibited a fluctuating Rg, with a rise from 14.8 to 14.9 Å, while for the Akt-C complex, Rg values exhibited a similar pattern and increased from 14.8 to 14.94 Å (Figure 6C). However, the Akt-C-P complex exhibited a lowering of the Rg peak from 14.9 to 14.8 Å. The Rg rise from the beginning to the end of the simulation signifies less compactness, while the lowering of the peak signifies greater compactness of the complex of the protein in the ligand-bound state. The number of H-bonds between protein and ligand signifies stable interaction between them. The complexes of Akt with both the ligands in isolation as well as in combination showed the formation of a significant number of H-bonds throughout the simulation time of 100 ns (Figure 6D). A consistent average of two H-bonds was observed between the protein and ligands in all the interacting events between Akt and the ligands, suggesting stable conformations of the complexes.

The FEL of attaining global minima of Cα backbone atoms of Akt with regard to RMSD and Rg are displayed in Figure 6E, with the deep blue basin indicating the achievement of global minima at respective RMSD and Rg values. Akt-P achieved the global minima (lowest free energy state) at RMSD 3.9 Å and Rg 20.8 Å (Figure 6E(1)). The Akt bound with curcumin showed global minima at RMSD 3.3 Å and Rg 20.7 Å (Figure 6E(2)). Interestingly, the global minima of Akt bound to the consortium of plumbagin and curcumin (Akt-C-P) were achieved at RMSD 2.9 Å and Rg 20.2 Å (Figure 6E(3)). The FEL anticipated a deterministic behavior of Akt at the lowest-energy state due to its high stability and optimal conformation in the ligand-bound states. The FEL, thus, is an indicator of the protein folding needed to acquire the lowest-energy state, which is largely attained at the Akt-C-P complex state.

**Figure 7:**
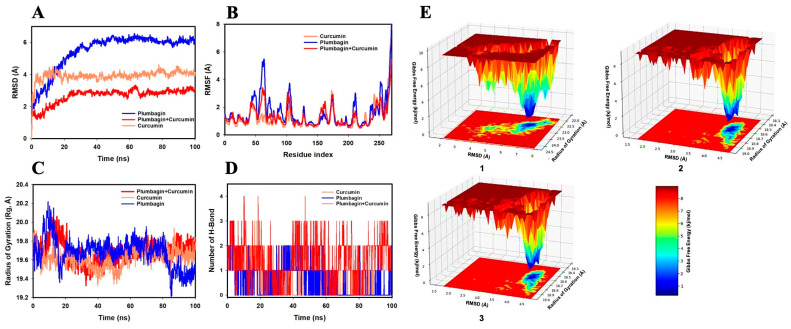
Analysis of MD simulation trajectories of 100 ns time scale for the interaction of curcumin and plumbagin with mTOR. (**A**) RMSD plot displaying the molecular vibration of Cα backbone of mTOR-P (blue), mTOR-C (orange), and mTOR-C-P (red). (**B**) RMSF plot showing the fluctuations of respective amino acids throughout the simulation time of 100 ns for mTOR-P (blue), mTOR-C (orange), and mTOR-C-P (red). (**C**) Rg plot for the deduction of compactness of mTOR-P (blue), mTOR-C (orange), and mTOR-C-P (red). (**D**) Number of H-bonds formed between mTOR and plumbagin (blue), mTOR and curcumin (orange), and mTOR and curcumin–plumbagin combination (red). (**E**) The FEL displays the progression of global minima (ΔG, kJ/mol) of mTOR in presence of (**1**) plumbagin, (**2**) curcumin, and (**3**) curcumin–plumbagin combination with respect to their RMSD (Å) and Rg (Å). The lower right panel displays the energy scale used in the measurement of FEL.

The RMSD values of the Cα backbone of mTOR-P and mTOR-C were found to be 6.1 Å and 4.0 Å, respectively, in the MD simulation analysis (Figure 7A). The complex mTOR-C-P formed when both curcumin and plumbagin were bound to the mTOR protein and had an RMSD of 2.0 Å. The lowering of RMSD in the mTOR-C-P indicates good convergence and stable conformation of the complex [25,26,27]. The RMSF plot showed considerable spikes of fluctuation in Cα-atoms of mTOR-P and mTOR-C complexes, whereas no substantial spikes were observed in the mTOR-C-P complex. Higher residual fluctuations were observed at the residues 47–65, 110–125, and 210–220. The increased residual fluctuations might be attributed to the higher flexibility of the residues conformed into the loop area, whereas the remainder of the residues fluctuated less during the whole of the 100 ns simulation, suggesting rigid amino acid conformations during the simulation duration (Figure 7B). Thus, the RMSF plots of Akt showing the interaction of mTOR with the combination of ligands indicate a rigid protein structure during the simulation [26,27]. Moreover, the Cα backbone of the mTOR-P complex exhibited a fluctuating Rg from 19.7 to 19.4 Å, while for mTOR-C, the Rg values exhibited a similar pattern and slightly decreased from 19.7 to 19.6 Å (Figure 7C). However, the mTOR-C-P complex exhibited a stable Rg peak ranging from 19.7 to 19.7 Å. The stable Rg peak signifies more compactness of the protein complex in the ligand-bound state. Furthermore, the number of H-bonds formed between a protein and a ligand contributes to the stability of the interaction. Throughout the simulation period of 100 ns between mTOR and the ligands, the formation of a steady number of H-bonds was observed. On average, the mTOR-P, mTOR-C, and mTOR-C-P complexes formed one, two, and three H-bonds, respectively (Figure 7D).

Figure 7E shows the FEL used for achieving global minima of the C backbone atoms of mTOR with respect to RMSD and Rg, with the deep blue basin denoting the accomplishment of global minima at the corresponding RMSD and Rg values. At an RMSD of 6.5 Å and Rg of 22.5 Å, mTOR-P reached the global minimum (Figure 7E(1)). The curcumin-bound mTOR had a global minimum at RMSD 4.0 Å and Rg 18.4 Å (Figure 7E(2)). It is interesting to note that the global minimum of mTOR bound to the combination of plumbagin and curcumin (Akt-C-P) was reached at an RMSD 3.9 Å and Rg 18.6 Å (Figure 7E(3)). Thus, the FEL is an indicator for protein folding needed to achieve the lowest-energy state, which was mostly accomplished at the mTOR-C-P complex state.

### 2.3. MM-GBSA Analysis of Curcumin and Plumbagin Synergistic Targeting of PI3K/Akt/mTOR

Molecular mechanics combined with the generalized Born surface area (MM-GBSA) is a reliable technique used to assess docking poses, assess structural stability, and predict binding affinities and hotspots. For each of the protein–ligand complexes of PI3K (PI3K-P, PI3K-C, and PI3K-C-P), Akt (Akt-P, Akt-C, and Akt-C-P), and mTOR (mTOR-P, mTOR-C, and mTOR-C-P), the binding free energy, as well as additional contributing energy in the form of MM-GBSA, were calculated. The results suggested that the greatest contributions to ΔG_bind_ in the stability of the simulated complexes were attributed to ΔG_bind_Coulomb, ΔG_bind_vdW, and ΔG_bind_Lipo, while ΔG_bind_Covalent and ΔG_bind_SolvGB contributed to the instability of the corresponding complexes (Table 2). When compared to the proteins that were just bound to plumbagin or curcumin, the PI3K-C-P, Ak-C-P, and mTOR-C-P complexes displayed much greater levels of binding free energy. These findings confirmed that plumbagin and curcumin might target the PI3K/Akt/mTOR pathway more effectively together than separately, supporting the possibility of a synergistic impact.

## 3. Materials and Methods

### 3.1. Preparation of Target Proteins

Protein Data Bank (https://www.rcsb.org/) was accessed on 1 March 2022 to obtain the 3D structure of the target proteins, PI3K (PDB ID: 5JHB), Akt (PDB ID: 3MV5), and mTOR (PDB ID: 4JSV). After removing water molecules and heteroatoms, adding polar hydrogen, and assigning Kollman charges to the receptor protein, it was ready for molecular docking studies [28]. 

### 3.2. Preparation of Ligands

The structures of the ligands curcumin and plumbagin chosen for this study were obtained from the NCBI PubChem (https://pubchem.ncbi.nlm.nih.gov/) database accessed on 1 March 2022. All atomic coordinates were transformed to .pdb format by using Open Babel [29]. They were further converted into .pdbqt format once the torsion angles and rotatable bonds were selected in the PDB structures.

### 3.3. Molecular Docking

For this study, both individual and sequential docking strategies were performed using AutoDock 4.2 software to study the synergistic interaction of the ligands with the target proteins [30]. The ligand-binding sites in sequential docking studies may differ from the protein’s traditional binding sites [24,31]. Therefore, we first performed blind docking for each protein by placing the entire protein inside a grid box. The potential binding poses were explored against each of the two ligands separately. Following the analysis of the binding areas of two compounds, sequential docking was used to determine the effect of these two compounds in combination with the protein. We used this method to see if there is any synergistic or antagonistic effect between the two ligands when allowed to interact with the same protein simultaneously [24]. The Lamarckian Genetic Algorithm was applied for molecular docking with the previously described parameters [28,32]. The protein–ligand interactions were then analyzed by using BIOVIA Discovery Studio.

In the beginning, plumbagin was docked to the PI3K protein’s best binding sites; the complex was given the name PI3K-P. Following this, curcumin was docked with PI3K-P and given the name PI3K-P-C. In a similar way, curcumin was also docked to the PI3K protein’s best binding sites. The complex was given the name PI3K-C. Plumbagin was then docked with PI3K-C and given the label PI3K-C-P. 

For the target Akt, the plumbagin was docked to its best binding sites of the Akt protein. Akt-P was the name given to the complex. Following this, curcumin was docked with Akt-P and given the name Akt-P-C. Similarly, curcumin was docked to its best binding sites of Akt protein. Akt-C was the name given to the complex. Following this, plumbagin was docked with Akt-C and given the name Akt-C-P. 

Finally, plumbagin was docked to the optimal binding sites on the mTOR protein, and the complex was given the designation mTOR-P. Curcumin was docked with mTOR-P and given the name mTOR-P-C as a result. Curcumin was docked to the optimal binding sites of the mTOR protein in a similar way. The complex was named mTOR-C. Plumbagin was subsequently docked against the mTOR-C complex as a target and named mTOR-C-P. 

### 3.4. MD Simulation

The MD simulations studies were performed on the docked complexes of PI3K, Akt, and mTOR, with ligands curcumin, plumbagin, and a consortium of curcumin and plumbagin using the Desmond 2020.1 from Schrödinger, LLC, Portland, OR, USA. In this system, the OPLS-2005 force field and explicit solvent model with SPC molecules were utilized in a period boundary solvation box of 10 Å × 10 Å × 10 Å dimensions [33,34,35]. To neutralize the charge of 0.15 M, Na^+^ ions were added, and a NaCl solution was supplied to the system to simulate the physiological condition. Initially, the system was equilibrated using an NVT ensemble for 10 ns to retrain over the protein–ligand complexes of PI3K (PI3K-P, PI3K-C, and PI3K-C-P), Akt (Akt-P, Akt-C, and Akt-C-P), and mTOR (mTOR-P, mTOR-C, and mTOR-C-P). Then, a quick run of equilibration and minimization was performed using NPT ensemble for 12 ns. The Nosé–Hoover chain coupling scheme was used to set up the NPT ensemble [36]. The temperature was set at 27 °C, the relaxation period set at 1.0 ps, and the pressure was held constant at 1 bar throughout all simulations. The time step was 2 fs. With a relaxation duration of 2 ps, the Martyna–Tuckerman–Klein chain coupling scheme barostat method was used for pressure control [37]. The long-range electrostatic interactions were calculated using the particle mesh Ewald method with the radius for the coulomb interactions set at 9 Å [38]. To compute the bonded forces, the RESPA integrator was used with a time step of 2 fs for each trajectory. Geo_measures v 0.8 was used to calculate the free energy landscape of protein folding on the ligan-bound complexes [39]. Geo_measures contain a strong g_sham library that records the MD trajectory against RMSD and Rg energy profile of folding in a 3D display using matplotlib python package. The final production run was conducted for 100 ns. The RMSD, Rg, RMSF, number of hydrogen bonds (H-bonds), and FEL were computed to monitor the stability of the MD simulations.

### 3.5. Binding Free Energy Analysis

The binding free energies of the ligand-bound complexes of PI3K (PI3K-P, PI3K-C, and PI3K-C-P), Akt (Akt-P, Akt-C, and Akt-C-P), and mTOR (mTOR-P, mTOR-C, and mTOR-C-P) were calculated using the MM-GBSA approach. The MM-GBSA binding free energy was computed in the simulation trajectory for the last 50 frames with a 1-step sample size using the Python script thermal mmgbsa.py. The binding free energy of Prime MM-GBSA (kcal/mol) was calculated using the principle of additivity, which included individual energy modules, such as columbic, covalent, hydrogen bond, van der Waal’s, lipophilic, and solvation, of ligand and protein. The following equation was used to calculate Δ*G_bind_*:

$$\Delta G_{bind}=\Delta G_{MM}+\Delta G_{Solv}-\Delta G_{SA}$$

where

- Δ*G_bind_* denotes the binding free energy;
- Δ*G_MM_* denotes the difference between the free energies of ligand–protein complexes and the total energies of protein and ligand in isolation;
- Δ*G_Solv_* denotes the difference in the GSA solvation energies of the ligand–protein complex and the sum of the solvation energies of the protein and the ligand in the unbound state;
- Δ*G_SA_* denotes the difference in the surface area energies for the protein and the ligand.

## 4. Conclusions

PI3K/Akt/mTOR signaling acts as a pro-survival mechanism for cancer cells and is found to be overexpressed in several types of cancer. Therefore, it provides an excellent target for novel anticancer therapeutic candidates. Curcumin and plumbagin are promising anticancer drug candidates that potentially suppress the PI3K/Akt/mTOR pathway that causes cancer cell death. The findings of this study report for the first time that when these two compounds are used in combination, they show a greater inhibitory effect on the PI3K/Akt/mTOR pathway than the individual compounds, indicating their synergistic potential. However, this study is entirely based on an in silico method; therefore, further studies in different cancer in vitro and in vivo models are required to assess the actual potency of curcumin and plumbagin consortium and enhanced anticancer efficacy in a synergistic manner. Further, the potential toxicity of the individual compound and their combinations also needs to be assessed.

## Figures and Tables

**Table 1:** The binding energy (ΔG), inhibition constant (Ki), and molecular interactions of plumbagin and curcumin sequentially docked against PI3K, Akt, and mTOR.

Sl. No.	Target	Ligand	Docking Complex	ΔG (kcal/mol)	Ki (μM)	Interacting Residues
1	PI3K	Plumbagin	PI3K-P	−6.44	19.06	Leu661, Phe694, Phe698, Met842, Leu845, Gln846, Arg849, Gly868, Cys869, Tyr787
2	PI3K	Curcumin	PI3K-C	−9.05	0.23	Gln291, Trp201, Asp654, Leu657, His658, Leu660, Leu661, Phe694, Phe698, Arg690, Tyr787, Met842, Leu845, Gln846, Arg849, Tyr867, Gly868, Cys869
3	PI3K-Plumbagin complex (PI3K-P)	Curcumin	PI3K-P-C	−9.24	0.17	Pro810, Trp812, Ile831, Lys833, Asp836, Leu838, Asp841, Ile879, Glu880, Ile881, Val882, Lys883, Asp884, Ala885, Thr886, Tyr867, Met953, Phe961, Ile963, Asp964
4	PI3K-Curcumin complex (PI3K-C)	Plumbagin	PI3K-C-P	−6.73	11.63	His389, Pro424, Lys425, Trp598, Val604, Tyr608, Ser636, Asp637, Glu638, Asn639
5	Akt	Plumbagin	Akt-P	−6.06	35.98	Tyr272, Arg273, Asp274, Leu275, Cys310, Gly311, Thr312, Tyr315, Leu316, Ala317, Val320, Val330, Asp331, Gly334
6	Akt	Curcumin	Akt-C	−8.31	0.80	Leu156, Gly157, Lys158, Gly159, Thr160, Phe161, Gly162, Lys163, Val164, Lys179, Met227, Gly233, Glu234, Phe237, Met281, Thr291, Asp292, Tyr437, Phe438, Asp439, Phe442
7	Akt-Plumbagin complex (Akt-P)	Curcumin	Akt-P-C	−9.24	0.17	Leu156, Gly157, Val164, Ala177, Lys179, Glu198, Leu202, Thr211, Met227, Glu228, Tyr229, Ala230, Glu234, Phe237, Met281, Thr291, Asp292, Phe293, Tyr437, Phe438, Asp439, Phe442
8	Akt-Curcumin complex (Akt-C)	Plumbagin	Akt-C-P	−5.32	126.77	Gly157, Lys158, Gly159, Val164, Glu234, Lys276, Glu278, Asn279, Asp292
9	mTOR	Plumbagin	mTOR-P	−5.09	187.26	Asn2206, Leu2209, Ala2210, Ser2215, Leu2216, Asn2219, Leu2220, Ser2221
10	mTOR	Curcumin	mTOR-C	−5.13	174.96	Tyr1787, His1791, Asn1898, Asn1899, Gln1901, Asp1902, Thr2207, Leu2208, Asn2211, Asp2212, Val2406, Glu2409, Pro2213, His2410, Ser2413
11	mTOR-Plumbagin complex (mTOR-P)	Curcumin	mTOR-P-C	−6.73	11.74	His1454, Trp1456, Glu1485, Ala1486, Ser1584, Tyr1587, Val1591, Gln1627, Arg1628, Ile1629, Glu1631, Asp1632, Lys1635
12	mTOR-Curcumin complex (mTOR-C)	Plumbagin	mTOR-C-P	−6.76	11.01	Leu2185, Lys2187, Tyr2225, Ile2237, Gly2238, Trp2239, Val2240, Met2345, Ile2356, Asp2357

**Table 2:** Binding free energy components for PI3K, Akt, and mTOR complexed with plumbagin, curcumin, and plumbagin–curcumin combination calculated by MM-GBSA.

Energies (kcal/mol)	PI3K-P	PI3K-C	PI3K-C-P	Akt-P	Akt-C	Akt-C-P	mTOR-P	mTOR-C	mTOR-C-P
**ΔG_bind_**	−42.07 ± 2.4	−38.35 ± 8.40	−76.93 ± 5.15	−61.42 ± 4.1	−66.04 ± 2.63	−85.26 ± 2.99	−42.58 ± 6.35	−41.04 ± 1.13	−56.81 ± 6.79
**ΔG_bind_Lipo**	−8.68 ± 2.44	−11.65 ± 2.08	−35.33 ± 2.61	−19.83 ± 2.3	−23.96 ± 1.03	−31.50 ± 3.1	−12.24 ± 1.23	−13.43 ± 1.6	−18.08 ± 1.04
**ΔG_bind_vdW**	−35.35 ± 6.44	−29.78 ± 6.17	−72.26 ± 5.40	−52.68 ± 2.17	−51.10 ± 2.0	−70.63 ± 2.63	−36.13 ± 2.17	−34.160 ± 3.0	−48.49 ± 2.18
**ΔG_bind_Coulomb**	−12.4 ± 2.21	−13.31 ± 8.69	−27.18 ± 3.11	−2.14 ± 1.01	−8.12 ± 1.99	−43.66 ± 2.88	−13.83 ± 5.54	−6.22 ± 0.99	−25.47 ± 6.20
**ΔG_bind_H_bond_**	−2.03 ± 0.77	−0.87 ± 0.6	−0.17 ± 0.1	−0.06 ± 0.01	−0.41 ± 0.22	−1.87 ± 0.5	−0.37 ± 0.2	−0.62 ± 0.16	−1.73 ± 0.34
**ΔG_bind_SolvGB**	8.15 ± 0.09	16.30 ± 7.42	58.35 ± 6.76	13.65 ± 2.27	16.5 ± 1.09	60.54 ± 2.8	19.73 ± 2.78	21.2 ± 1.7	31.74 ± 3.34
**ΔG_bind_Covalent**	2.01 ± 0.72	1.83 ± 1.28	3.27 ± 3.02	0.85 ± 0.5	1.56 ± 1.2	4.22 ± 1.07	2.97 ± 1.90	2.66 ± 1.12	5.23 ± 4.41

## Data Availability

The data will be available by corresponding authors upon genuine request.
